# An algorithm based on positive and negative links for community detection in signed networks

**DOI:** 10.1038/s41598-017-11463-y

**Published:** 2017-09-07

**Authors:** Yansen Su, Bangju Wang, Fan Cheng, Lei Zhang, Xingyi Zhang, Linqiang Pan

**Affiliations:** 1 Key Lab of Intelligent Computing and Signal Processing of Ministry of Education, School of Computer Science and Technology, Anhui University, Hefei, 230039 China; 2 Key Laboratory of Image Processing and Intelligent Control, School of Automation, Huazhong University of Science and Technology, Wuhan, 430074 China; 3 School of Electric and Information Engineering, Zhengzhou University of Light Industry, Zhengzhou, 450002 Henan China

## Abstract

Community detection problem in networks has received a great deal of attention during the past decade. Most of community detection algorithms took into account only positive links, but they are not suitable for signed networks. In our work, we propose an algorithm based on random walks for community detection in signed networks. Firstly, the local maximum degree node which has a larger degree compared with its neighbors is identified, and the initial communities are detected based on local maximum degree nodes. Then, we calculate a probability for the node to be attracted into a community by positive links based on random walks, as well as a probability for the node to be away from the community on the basis of negative links. If the former probability is larger than the latter, then it is added into a community; otherwise, the node could not be added into any current communities, and a new initial community may be identified. Finally, we use the community optimization method to merge similar communities. The proposed algorithm makes full use of both positive and negative links to enhance its performance. Experimental results on both synthetic and real-world signed networks demonstrate the effectiveness of the proposed algorithm.

## Introduction

Many complex systems in the real world can be modeled as networks^1^. The networks that include only positive links are called unsigned networks, and the networks with both positive and negative links are called signed networks. Compared with unsigned networks, the links in a signed networks bring more information. Specifically, a positive link in a unsigned network just means a ‘relationship’, while a positive link in signed networks denotes a ‘positive relationship’, and a negative one denotes a ‘negative relationship’. For example, in a signed social network, the relationships between parties may be political alliances and oppositions^2^. Ferligoj and Kramberger has established the positive links and the negative links to represent the political arrangements with positive and negative ties, respectively^2^. Besides, there are positive relationships–friendship, trust and like, as well as negative relationships–hostility, mistrust and dislike. In the field of biological science, a gene may be enhanced or repressed by another gene, and the enhanced or repressed relationships could be reflected by the positive or negative links^3–6^. A protein is likely to be expressed in a subtype of lung cancer, while it is unexpressed in another subtype of lung cancer. The relationships between proteins and the subtypes of lung cancer could also be reflected by positive and negative links^7^. Recently, Kunegis *et al*. showed that taking the positive and negative links into consideration could help to find more useful information compared with the only analysis of positive links^8^.

Community detection problem has attracted increasing attention since it was first proposed by Girvan and Newman^9^. Most of these community detection methods can only handle the networks without negative links, i.e. unsigned networks^9–19^. In an unsigned network, communities are defined as the groups of nodes in which links are dense, while between which are less dense. Unlike the definition above, the communities in signed networks are defined as the groups of nodes in which positive links are dense and between which negative links are also dense. That is, community detection methods in unsigned networks focus merely on link density but not the signs of links as their clustering attributes. However, the communities in signed networks depend on not only the density of links but also the signs of links. Thus, previous community detection algorithms in unsigned networks are not suitable for the community detection problem in signed networks. In view of the importance of signed networks, community detection methods in signed networks need to be developed. The challenge of the community detection problem in signed networks is that the community structure is ambiguous since that there are some negative links within communities and some positive links between communities. In the face of the challenge, researchers have put forward lots of community structure detection algorithms to get the best partition of signed networks.

Several algorithms have been extended from the community detection algorithm in unsigned networks to solve community detection problem in signed networks^20^. Yang *et al*. first proposed the FEC algorithm to detect communities from signed networks based on random walk. Subsequently, several two-stage clustering algorithms have been proposed^21–23^. For instance, the community modularity values are respectively calculated by positive and negative links, and the communities are evaluated by the combination of these two community modularity values^21^. The GN-H algorithm is the combination of GN and hierarchical clustering algorithm to detect communities in signed networks^22^. Specifically, it uses the GN algorithm to detect communities based on the positive links, and then combine the negative links to get the final hierarchical clustering results. However, in these two-stage clustering algorithms, the latter stage is always affected by the previous stage, which may limit the performance of the algorithms. Liu *et al*. first proposed the community detection problem as a multiobject problem (MOP), but the proposed objective functions still need further optimization and improvement to enhance its performance^24^. Majority of previous researches mainly use positive links for community detection, and negative links are only used for adjustment. In fact, positive links attract a node to be in a community, while the node is rejected outside by negative links. Negative links have no less information than positive links. Thus, further study is needed to make full use of both positive and negative links for community detection in signed networks.

In our work, we propose a random walk-based algorithm named SRWA for community detection in signed networks based on positive and negative links. The overall framework of SRWA is to detect initial communities in a signed network, and then expand these initial communities by application of random walks. Firstly, a dense subgraph is detected based on the nodes, whose degree is larger than that of its neighbours. Then, the initial community is growing by adding the node which is more likely to be attracted into the community than to be rejected from the community step by step. Specifically, a node which is not in a current community has a positive probability to be in the community and a negative probability to be away from the initial community. The positive probability is compared with the negative one to judge whether the node should be added into the community. If a node could not be added into current communities, then a new initial community may be developed. Experimental results on both synthetic and real-world signed networks show the feasibility and effectiveness of the proposed algorithm.

## Results

In this section, we present the comparative results of the proposed algorithm and the representative algorithms, i.e., FEC^20^, MEAs-SN^24^ and a method to optimize the modularity based on Tabu search which is implemented by Radatool (Tabu search for short)^21, 25^, on both real-world and synthetic signed networks.

### Real-world and synthetic signed networks

#### Real-world signed networks

**Figure 1:**
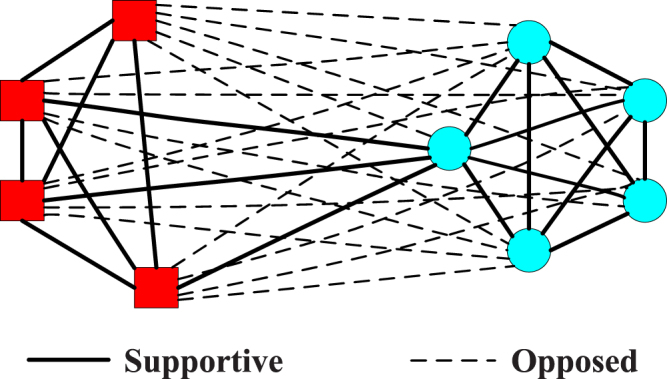
The U.S. supreme court justices network.

The first real social network is the U.S. supreme court justices network, which describes the voting behavior of nine justices in the supreme court of the United States during the period of 2006–2007^26^. The positive line means that one justice supports the other one, and the negative line indicates the opposite meaning. Its community structure is shown in Fig. 1. We can see that the U.S. supreme court justices network is divided into two communities.

**Figure 2:**
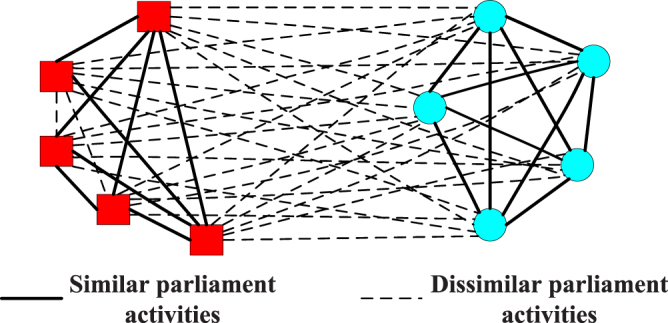
The Slovene parliamentary party network.

The Slovene parliamentary party network represents the relationships among ten parties of the Slovene parliamentary in 1994^2^. Positive links mean that the parliament activities of two parities are similar, while negative links mean that their activities are dissimilar. Figure 2 shows the topological structure of the Slovene parliamentary party network and its community structure.

**Figure 3:**
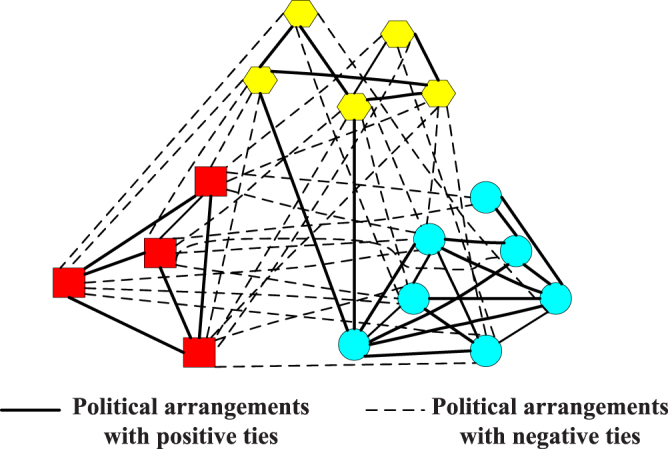
The Gahuku-Gama subtribes network.

The Gahuku-Gama subtribes network reflects the political alliances and oppositions among 16 Gahuku-Gama subtribes, which are distributed in a particular area and are involved in warfare with each other^27^. Positive and negative links represent the political arrangements with positive and negative ties, respectively. Its community structure can be seen in Fig. 3.

The Sampson monastery network represents the social relationships between 18 monks in the monastery of new England^28^. Sampson collected four kinds of social relationships among a group of monks, i.e., friendship, esteem, influence and sanction. Each type of relationship has both positive and negative aspects. Six variants of the Sampson monastery network can be obtained from UCINET IV datasets and each variant consists of 18 nodes, however, the numbers of positive and negative links are different in these variants. The information about the six variants of the Sampson monastery network are described in Table 1 ^29^. All these variants have three communities due to the fact that 18 monks were divided into three groups, i.e., Young Turks, Outcasts, and Loyal Opposition^29^.

**Table 1:** Six variants of Sampson Monastery Network^29^.

Name of signed networks	(*N*_*p*_, *N*_*n*_)	Relationships	Attributes of relationship	
			Positive	Negative
SAM-AFF4	(56, 47)	friendship	like	dislike
SAM-AFF3	(57, 48)	friendship	like	dislike
SAM-AFF2	(55, 49)	friendship	like	dislike
SAM-EST	(54, 58)	esteem	esteem	disesteem
SAM-INFL	(53, 50)	influence	positive	negative
SAM-SANC	(39, 41)	sanction	praise	blame

‘(*N*_*p*_, *N*_*n*_)’ denotes that the number of positive links in a network is *N*_*p*_, and that of negative links is *N*_*n*_.

The microarray expression data for the construction of a gene network used in the study originated from the Gene Expression Omnibus (GEO) with the accession number GSE23400 (http://www.ncbi.nlm.nih.gov/). There are 52 samples and each sample contains expression data of 54,675 probes, which are associated to genes according to the information of GPL570 (a microarray chip). According to the number of genes that a probe detects, probes can be classified into three categories: probes detecting a single gene, probes detecting more than one gene, and probes detecting no genes. We performed the removal of probes which could not detect any genes in each sample, and calculated the expression value of each gene which could be detected by more than one probe. In addition, we calculated the Pearson correlation coefficients of two genes based on their expression data. If the Pearson correlation coefficient between *gene*_1_ and *gene*_2_ is larger than 0.8 or smaller than −0.8, then a positive link or a negative link is considered between *gene*_1_ and *gene*_2_. A positive link between *gene*_1_ and *gene*_2_ denotes that *gene*_1_ and *gene*_2_ are positively related, and a negative link means that they have a negative correlation. Then, a gene-gene interaction network (GIN, for short) is constructed, including 658 nodes and 3338 links, where 2774 are positive links and 564 are negative links.

#### Synthetic signed networks

In this work, we extended the Lancichinetti-Fortunato-Radicchi (LFR, for short) benchmark to signed networks^30^. A signed network generator is designed with an unsigned network generator and a program to control the type of links in an unsigned network^31^. The signed network generator is denoted as *SRN*(*n*, *k*, *maxk*, *t*_1_, *t*_2_, *minc*, *maxc*, *on*, *om*, *μ*, *P*_−_, *P*_+_). Here, *N* is the number of nodes in a network; *k* and *maxk* are the average and maximum degree of nodes; *t*_1_ and *t*_2_ are the exponents for the degree and community size distribution; *mimc* and *maxc* are the minimum and maximum community size; *on* and *om* are the number of overlapping nodes and the number of memberships of overlapping nodes. More importantly, *μ* is the fraction of links that each node shares with nodes in other communities, which controls the cohesiveness of the communities in the generated SRNs. The higher the value of *μ* is, the more ambiguous the community structure is. *P*_−_ is the fraction of negative links within communities, while *P*_+_ is the fraction of positive links between communities. Ideally, negative links should be between communities and positive links should be within communities. Thus, *P*_−_ and *P*_+_ are two parameters to adjust the noise level. When the value of *μ* is fixed, the larger the values of *P*_−_ and *P*_+_ are, the more ambiguous the community structure is. That is, given a fixed *μ*, we can control the noise level by adjusting both *P*_−_ and *P*_+_. In this experiment, we produce three groups of signed LFR benchmark networks. All groups share parameters *maxk* = 50, *t*_1_ = 2, *t*_2_ = 1, *minc* = 10 and *maxc* = 30. The values of other parameters show differences in different groups. One group contains 100 networks, which share the parameters *N* = 128, *k* = 16; *μ* increases from 0.1 to 0.5 in the step of 0.1; *P*_+_ increases from 0.0 to 0.8 in the step of 0.2; *P*_−_ increases from 0.0 to 0.6 in the step of 0.2. Each of the other two groups contains 12 networks. These two groups share parameter *k* = 10, *μ* ∈ {0.3, 0.5}, *P*_+_  ∈ {0.1, 0.3, 0.5}, and *P*_−_ ∈ {0.1, 0.3}. The number of nodes is set to be 500 and 1000 in these two groups, respectively. The detailed information about each group is shown in Table 2.

**Table 2:** Information of LFR benchmark signed networks.

Group name	N	k	*μ*	*P*_+_	*P*_−_
Group 1	128	16	0.1–0.5	0.0–0.8	0.0–0.6
Group 2	500	10	0.3–0.5	0.1–0.5	0.1–0.3
Group 3	1000	10	0.3–0.5	0.1–0.5	0.1–0.3

‘*N*’ represents the number of nodes in a network; ‘*k*’ denotes the average degree of nodes; *μ* is the fraction of links that each node shares with nodes in other communities; *P*_−_ is the fraction of negative links within communities, while *P*_+_ is the fraction of positive links between communities.

### Comparison with other algorithms

We verify the performance of the proposed algorithm (SRWA) by comparing it with three representative algorithms (FEC, MEAs-SN, and Tabu search) on both real-world and synthetic signed networks.

#### Comparison on real-world signed networks

As can be seen in Table 3, the proposed algorithm could generate the true partition results on the networks (e.g., the U.S. supreme court justices network, the Slovene parliamentary party network, the Gahuku-Gama subtribes network, and two variants (i.e., SAM-AFF4 and SAM-INFL) of the Sampson monastery networks). Besides, the obtained *NMI* and *Q*_*signed*_ values were almost larger than those of other algorithms.

**Table 3:** The values of *NMI* and *Q*_*signed*_ on real-world networks.

Networks	*NMI*				*Q*_*signed*_			
	FEC	MEAs-SN	Tabu	SRWA	FEC	MEAs-SN	Tabu	SRWA
SCJ	**1**	**1**	**1**	**1**	**0.4050**	**0.4050**	**0.4050**	**0.4050**
SPP	0.8572	0.8483	**1**	**1**	0.4086	0.4022	**0.4547**	**0.4547**
GGS	0.9143	0.9022	**1**	**1**	0.3870	0.3779	**0.4310**	**0.4310**
SAM-AFF4	0.7007	0.7492	**1**	**1**	0.3039	0.2261	**0.3969**	**0.3969**
SAM-INFL	0.5296	0.7448	**1**	**1**	0.1460	0.1839	**0.3604**	**0.3604**
GIN	—	—	—	—	0.2220	0.1827	**0.4577**	0.2901

‘SCJ’, ‘SPP’, and ‘GGS’ represent the U.S. supreme court justices network, the Slovene parliamentary party network, the Gahuku-Gama subtribes network, respectively. ‘SAM-AFF4’ and ‘SAM-INFL’ are two variants of the Sampson monastery network. ‘GIN’ denotes the gene-gene interaction network. ‘Tabu’ represents Tabu search. ‘–’ means a null value.

We also examined the performance of the proposed algorithm on the gene-gene interaction network, the truth partition of which is unknown. Although the *Q*_*signed*_ value of the proposed SRWA (i.e., 0.2901) was smaller than that of Tabu search (i.e., 0.4577) on the gene-gene interaction network, the communities achieved by SRWA seem to be more reasonable than those obtained by Tabu search and other compared algorithms. To be specific, on the gene-gene interaction network, SRWA detected 41 communities, among which 11 communities were confirmed to be related to certain biological processes by the database for annotation, visualization and integrated discovery (DAVID for short, https://david.ncifcrf.gov/summary.jsp) (see Table 4). For example, a community detected by SRWA contains seven nodes, which represent the genes *ANKH*, *RP4-758J24.5*, *MIR6741*, *DNAJC30*, *NEIL2*, *NSMAF* and *XRN2*, respectively. Interestingly, above seven genes are all phosphoproteins, which are bound to phosphoric acid. In addition, the other ten communities detected by SRWA are corresponding to the following biological functions: membrane, alternative splicing, splice variant, protein binding, signal peptide, sequence variant, splice variant and cytoplasm. Here, we refer to a community which is confirmed to be related to a biological process by DAVID as an effective community. The ratio of the effective communities to all communities detected by SRWA is 0.268. However, the ratios of the effective communities to all communities detected by the compared algorithms (FEC, MEAs-SN and Tabu search) are respectively 0.017, 0.004 and 0.022, which are smaller than that by SRWA. Therefore, the SRWA performed better than other compared algorithms on the gene-gene interaction network.

**Table 4:** The effective communities on the gene-gene interaction networks.

Algorithms	FEC	MEAs-SN	Tabu	SRWA
*N*_*a*_	227	416	217	41
*N*_*e*_	4	2	6	11
$\frac{{N}_{e}}{{N}_{a}}$	0.017	0.004	0.022	0.268

‘*N*_*a*_’ represents the total number of detected communities. ‘*N*_*e*_’ denotes the number of effective communities among all detected communities. ‘Tabu’ represents Tabu search.

#### Comparison on synthetic signed networks

All algorithms are tested on three groups of synthetic signed networks. A total of 30 independent runs are conducted for each algorithm and the average results are shown.

1. Comparison results on synthetic signed networks with 128 nodes

**Figure 4:**
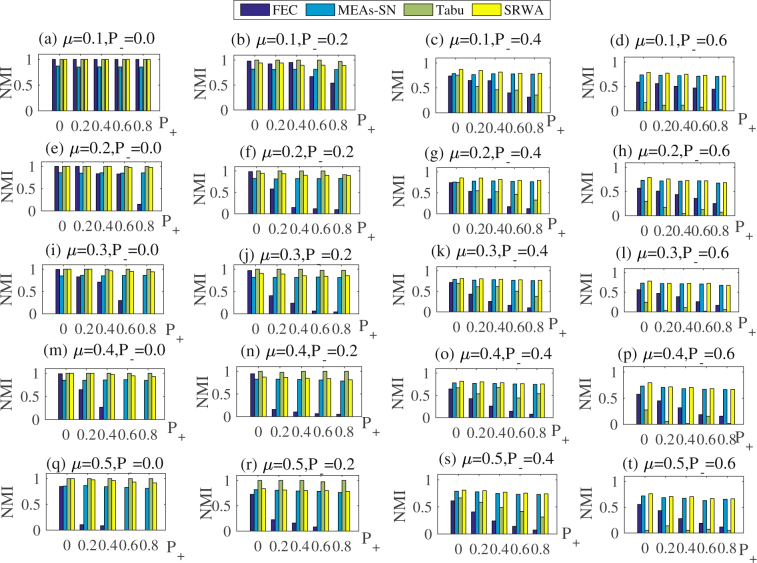
Comparison between SRWA and other algorithms on synthetic signed networks with 128 nodes.

As can be seen from Fig. 4(a,b,e,f,i,j,m and n), when the parameter *P*_−_ ≤ 0.2, the *NMI* obtained by the proposed SRWA is larger than that obtained by MEAs-SN, but it is smaller than that obtained by FEC or Tabu search for few detection problems, which suggests that the performance of SRWA is not the best on all synthetic signed networks. However, in these situations, the *NMI* obtained by SRWA is larger than 0.90, meaning that SRWA could get nearly true partition results. For example, when *μ* = 0.1 and *P*_−_ = 0, the *NMI* obtained by the proposed algorithm is always 1, as *P*_+_ increases from 0 to 0.8 (Fig. 4(a)). It suggests that in this situation SRWA could get the completely true partition results. In addition, the performance of SRWA is still better than that of FEC in term of stability. To be specific, for FEC, its performance decreases obviously with the increasing of *μ*, *P*_+_ and *P*_−_. For instance, when *μ* = 0.2 and *P*_−_ = 0.2, the value of *NMI* largely decreases when *P*_+_ increases from 0 to 0.8. Similarly, when *μ* = 0.1 and *P*_+_ = 0.2, the increase of *P*_−_ causes huge drops in the performance of FEC. If the values of *P*_+_ and *P*_−_ are both fixed, the value of *NMI* decreases with the increase of the *μ* value. It means that FEC is very sensitive to the parameters *μ*, *P*_+_ and *P*_−_. That is because there are some uncertain factors which lead to the instability of FEC, such as the random selection of the initial starting node. Although the increase of *μ*, *P*_−_, and *P*_+_ may also cause the decline of *NMI* by SRWA, there is a smaller decrease by SRWA than by FEC (Fig. 4).

When the parameter *P*_−_ > 0.2, the *NMI* value of SRWA is larger than those of other algorithms Fig. 4(c,d,g and h). For the Tabu search, despite it achieves the largest *NMI* when *P*_−_ ≤ 0.2, the increase of *P*_−_ causes huge drops of *NMI*. For example, when *μ* = 0.1 and *P*_−_ = 0.6, the performance of Tabu search in term of *NMI* is smaller than 0.3. However, in the situation, the value of *NMI* obtained by SRWA is larger than 0.75. Thus, SRWA performs better than Tabu search when *P*_−_ > 0.2. It may due to the fact that Tabu search is based on the maximization of modularity, which shows less effective when the community structure is unclear. That is to say SRWA shows its superior performance on signed networks with unclear community structures.

(2) Comparison results on synthetic signed networks with 500 and 1000 nodes

**Figure 5:**
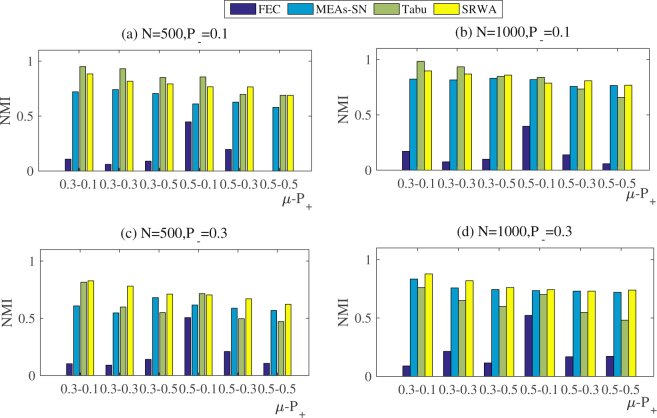
Comparison between SRWA and other algorithms on synthetic signed networks with 500 and 1000 nodes.

We also test the performance of SRWA on the synthetic signed networks with 500 and 1000 nodes. According to Fig. 5(a and b), we can see that when *P*_−_ = 0.1 the *NMI* obtained by SRWA is no less than 0.8, and in few situations it is smaller than that achieved by the Tabu search. It suggests that SRWA performs slightly less well than Tabu search for few detection problems, which is similar to the results on the synthetic networks with 128 nodes. In addition, on these two group of synthetic signed networks we find that SRWA may achieve larger *NMI* values than Tabu search when *μ* or *P*_+_ is larger, e.g., *μ* = 0.3 and *P*_+_ = 0.5 or *μ* = 0.5 and *P*_+_  ∈ {0.3,0.5} (Fig. 5(b)). That is to say, when *P*_−_ = 0.1, as the increase of *μ* or *P*_+_, SRWA shows its superior to Tabu search in terms of *NMI*.

In addition, SRWA almost performs the best when the parameter *P*_−_ = 0.3 (Fig. 5(c) and (d)). It is concluded that the performance of SRWA is superior to the comparative algorithm on the benchmark networks with 500 and 1000 nodes.

## Discussion

In this work, we have proposed a new algorithm, named SRWA, for detecting community structures in signed networks. The key component of SRWA is that a node which is not in any current communities may be added into a community on the basis of random walks, which makes full use of both positive and negative links between the node and the members of a community. We have tested the performance of SRWA, and compared it with other representative algorithms (FEC, MEAs-SN and Tabu search) on both real-world and synthetic signed networks. The experimental results have demonstrated the feasibility and effectiveness of SRWA. The feature of the proposed algorithm could be summarized as follows. (1) SRWA has a good ability to detect communities on signed networks. Several other algorithms have good performances on small-scale networks with clear community structures, however, their detection results are far from the expectation on large-scale networks with unclear community structure. The proposed SRWA shows its superiority over the competing approaches for detecting communities in signed networks with unclear community structures in terms of the quality of found communities. (2) SRWA is not sensitive to the initial nodes and it needs not any prior knowledge on the community structure.

In our future work, we will focus on how we can use the SRWA approach to further address problems in other related domains such as disease module mining. So far, the work about disease module mining considers a biological network as a large graph including only positive links. However, the relations among the entities of the biological network are complex, which could not be modeled only by positive links. From such a signed biological network, we may discover some previously unknown information. In addition, it is also interesting to investigate bio-inspired computing models for community detection in complex networks, such as probe machine^32^ and spiking neural P system^33^.

## Methods

A signed network can be abstracted as a graph *SN* = (*V*_*G*_, *E* ^*P*^, *E* ^*N*^), where *V*_*SN*_ = {*v*_1_, *v*_2_, …, *v*_*n*_} is the set of nodes in the network, *E* ^*P*^ is the set of positive links and *E* ^*N*^ is the set of negative links. The graph could be expressed as an adjacency matrix *A*, where the element *a*(*i*, *j*) represents the type of the link between the nodes *v*_*i*_ and *v*_*j*_ (i.e., < *v*_*i*_, *v*_*j*_ >). Specifically, if the link between the nodes *v*_*i*_ and *v*_*j*_ is positive, then *a*_*ij*_ = 1; if the link between *v*_*i*_ and *v*_*j*_ is negative, then *a*_*ij*_ = −1; if there is no relationships, then *a*_*ij*_ = 0.

The community detection in signed networks is to detect the communities in which the links are positive and between which the links are negative. Let *C* = {*C*_1_, *C*_2_, …, *C*_*m*_} be a set of communities in a signed network. The community detection problem in the signed network can be described as: *a*_*ij*_ = 1, (*v*_*i*_ ∈ *C*_*k*_)∧(*v*_*j*_ ∈ *C*_*k*_); *a*_*ij*_ = −1, (*v*_*i*_ ∈ *C*_*k*_) ∧ (*v*_*j*_ ∈ *C*_*l*_) ∧ (*l* ≠ *k*).

The proposed algorithm aims to make full use of both positive and negative links to detect communities in a signed network. The overall framework of SRWA is presented in Table 5, which consists of three main steps: (1) the initial communities are detected; (2) the initial communities are expanded based on random walks; (3) a procedure for community optimization is performed. In what follows, we introduce the details of SWRA.

**Table 5:** The overall framework of SRWA.

*Input*	Signed network *SN*;
*Output*	Community set *Y*.
Step 1	Calculate the node degree of each node in *SN* by Eq 1;
	Find the node which has a larger degree compared with its neighbors, and put it in set *H*.
	/* Each node in *H* is a local maximum degree node. */
Step 2	For each node *v*_*i*_ in set *H*, discover initial communities ${Y}^{{v}_{i}}$;
	Put the elements of ${Y}^{{v}_{i}}$ in set *Y*.
	/* *Y* is the set of initial communities. */
	/* ${Y}^{{v}_{i}}$ is the set of initial communities, in which each community contains *v*_*i*_. */
Step 3	Merge the initial communities which are identical in *Y*;
	Return *Y* as the set of initial communities;
	Put all nodes in initial communities in *V*;
	Put the rest nodes which are not in initial communities in *U*.
	/* *V* is the set of nodes which are in initial communities. */
	/* *U* is the set of nodes which are not in any initial communities. */
Step 4	For each node *u*_*i*_ in *U* and an initial community *Y*_*k*_ in *Y*, calculate *P* ^+^(*u*_*i*_ → *Y*_*k*_) and *P* ^−^(*u*_*i*_ → *Y*_*k*_) by Algorithm 1.
	/* *Y*_*k*_ is the *k* ^*th*^ community in *Y*. */
	/* *P* ^+^(*u*_*i*_ → *Y*_*k*_) means the positive probability of *u*_*i*_ belonging to *Y*_*k*_. */
	/* *P* ^−^(*u*_*i*_ → *Y*_*k*_) means the negative probability which represents *u*_*i*_ is away from *Y*_*k*_. */
Step 5	Compare *P* ^+^(*u*_*i*_ → *Y*_*k*_) with *P* ^−^(*u*_*i*_ → *Y*_*k*_);
	If *P* ^+^(*u*_*i*_ → *Y*_*k*_) > *P* ^−^(*u*_*i*_ → *Y*_*k*_), then put *k* in *Temp* − *number*.
	/* *Temp* − *number* is used to storage the number of initial community which is likely to contain *u*_*i*_. */
Step 6	If |*Temp* − *number*| ≠ 0, then add *u*_*i*_ to the community *Y*_*best*_ that results in the largest positive probability;
	/* *P* ^+^(*u*_*i*_ → *Y*_*best*_) = *maximum*{*P* ^+^(*u*_*i*_ → *Y*_*j*_)|*j* ∈ Temp-number } */
	Delete *u*_*i*_ form *U*.
Step 7	If |*Temp* − *number*| = 0, for *u*_*i*_, discover initial communities *Y* ^*u*^;
	If *Y* ^*u*^ − *Y* ≠ ∅, then *Y* = *Y* ∪ *Y* ^*u*^;
	Put all nodes included in *Y* in *V*;
	Put the rest nodes which are not included in *Y* into *U*.
Step 8	Repeat step 4–7 until there is no node left in *U*.
Step 9	Merge the communities which are identical or similar in *Y* by Eq. 14;
	Return *Y* as the set of communities.

### Detecting initial communities in signed networks

The node with a large impact in a network always has a large number of neighbours. The importance of a node could be reflected by the node degree, which is the sum of the positive degree and the absolute value of the negative degree (Eq. 1).

1 $$deg(v)=de{g}_{P}(v)+|de{g}_{N}(v)|,$$

where *deg*(*v*) represents the node degree, and *deg*_*P*_(*v*) and *deg*_*N*_(*v*) are the positive degree and the negative degree of the node, respectively. Specifically, if the degree of a node is larger than those of its neighbours, then the node is more likely to be a center of a community than its neighbours. The local maximum degree node is defined as the node which has a larger degree compared with its neighbors^13^. The way to discover the local maximum degree nodes was referred to the previous work^13^. In this work, we identify the local maximum degree nodes from all nodes in a signed network based on node degrees.

Here, a initial community in a signed network is defined as a dense subgraph, which includes a local maximum degree node, as well as its close neighbors. Given a local maximum degree node (*node*_1_), we identify its neighbour node (*node*_2_) with the largest positive degree. The reason why the positive degree of *node*_1_ is used to identify *node*_2_ is that, as members of initial communities, *node*_1_ and *node*_2_ should be linked closely by positive links. *node*_1_ and *node*_2_ may have a common neighbour node (*node*_3_), which is also detected based on positive degrees. A initial community is comprised by the nodes *node*_1_, *node*_2_ and *node*_3_, together with the links among them.

### Expanding communities

Let *Y* = {*Y*_*k*_|*k* = 1, …, *q*} be the set of all communities, where *q* is the number of the communities, ${Y}_{k}=({V}_{k},{E}_{k}^{P},{E}_{k}^{N})$ is the *k* ^*th*^ community, *V*_*k*_ is the set of nodes in the community, ${E}_{k}^{P}$ and ${E}_{k}^{N}$ are respectively the set of positive and negative links in the community. Specifically, in the initial situation, *Y*_*k*_(*k* = 1, …, *q*) is an initial community.

Let the walker start from a node *u*, which is not belong to any current communities. Then, the node *u* could teleport to current communities with probabilities on the basis of the connections of nodes. The total probability theorem and conditioning probability model are used to calculate the positive probability of the node *u* teleporting to a community based on positive links (i.e., *p* ^+^(*u* → *Y*_*k*_)(*k* = 1, …, *q*)), as well as the negative probability which represents the node is away from the community based on negative links (i.e., *p* ^−^(*u* → *Y*_*k*_)(*k* = 1, …, *q*)). If the positive probability of the node *u* teleporting to a community is larger than the negative probability of *u* being away from the community, then *u* may be added into the community; otherwise, it is not in current communities, which implies that a new initial community should be formed.

There are *q* initial communities, so we perform *q* runs of random walks to calculate *p* ^+^(*u* → *Y*_*k*_) and *p* ^−^(*u* → *Y*_*k*_). At the *k* ^*th*^ run of random walks, it is supposed that *u* belongs to the *k* ^*th*^ community. The graph of the *k* ^*th*^ random walk process is

2 $${G}_{k}=({V}_{k}^{^{\prime} },{E}_{k}^{P^{\prime} },{E}_{k}^{N^{\prime} }),$$

where $${V}_{k}^{^{\prime} }={\cup }_{t=1}^{q}\,{V}_{k}\cup \{u\}$$, $${E}_{k}^{P^{\prime} }=({\cup }_{t\mathrm{=1}}^{q}{E}_{k}^{P})\cup \{(u,{v}_{i})|{v}_{i}\in {V}_{k}\mathrm{,1}\le k\le q\}$$, $${E}_{k}^{N^{\prime} }={E}_{k}^{N}$$.

First, we calculate the positive and negative probability of the walker teleporting from *u* to the node *v*_*i*_(*i* = 1, …, *m*) in the graph *G*_*k*_. The way to calculate the positive and negative probability is the same except that they are based on different kinds of links.

Take the calculation of the positive probability of the walker teleporting from *u* to *v*_*i*_ for example. From the time *t* to *t* + 1, the walker has a teleporting probability *α* to jump, and a probability 1 − *α* to stay. Usually, the teleporting probability *α* is 0.15^34^. When the walker jumps, it may jump to a node with a transition probability. Suppose that the transition probability from *u* to *v*_*i*_ (*i* = 1, …, *m*) is the same, then the transition probability vector is $d={(\frac{1}{m},\frac{1}{m},\cdot \cdot \cdot ,\frac{1}{m})}^{T}$, where *m* is the number of the nodes in the *k* ^*th*^ community, and *d* is a *m* × 1 vector. When the walker stays, it may reach a node based on the positive similarity between nodes. The way to calculate the positive similarity between nodes is based on the positive links. Here, we make use of the similarity definition that Jaccard provided in the literature to evaluate the positive similarity between the nodes *v*_*i*_ ∈ *V* and *v*_*j*_ ∈ *V* (1 ≤ *i*, *j *≤ *m*) as follows^35–37^.

3 $$Simila{r}^{+}({v}_{i},{v}_{j})=\frac{|{{\rm{\Gamma }}}_{{v}_{i}}^{+}\cap {{\rm{\Gamma }}}_{{v}_{j}}^{+}|}{|{{\rm{\Gamma }}}_{{v}_{i}}^{+}|\cup |{{\rm{\Gamma }}}_{{v}_{j}}^{+}|},$$

where ${\Gamma }_{{v}_{i}}^{+}$ (${\Gamma }_{{v}_{j}}^{+}$) is the positive neighborhood of *v*_*i*_ (*v*_*j*_), the member of which is connected with *v*_*i*_ (*v*_*j*_) by a positive link, and |*x*| indicates the cardinality (i.e., number of elements) in the set *x*. Let *v*_*j*_ = *u* in Eq. 3. *Similar* ^+^(*v*_*i*_,*u*) represents the positive similarity between *u* and *v*_*i*_ ∈ *V* (1 ≤ *i *≤ *m*), and it is also denoted as *Similar* ^+^(*v*_*i*_) for short.

Let the matrix *M* ^+^ be the normalization of the positive similarity between nodes in the *k* ^*th*^ community. That is, ${M}^{+}(i,j)=\frac{Simila{r}^{+}({v}_{i},{v}_{j})}{{\sum }_{{v}_{j}}Simila{r}^{+}({v}_{i},{v}_{j})}$. Here, *M* ^+^ could be considered as the transition matrix of a random walker. Suppose the positive probability of the walker teleporting from *u* to *v*_*i*_ is ${s}_{t}^{+}(i)$ at the time *t*. Particularly, in the initial situation, the positive probability of the walker teleporting from *u* to *v*_*i*_ is the normalization of the positive similarity between *u* and *v*_*i*_, i.e., ${s}_{0}{(i)}^{+}=\frac{Simila{r}^{+}({v}_{i})}{{\sum }_{{v}_{i}}Simila{r}^{+}({v}_{i})}$. At the time *t* + 1, the positive probability ${s}_{t+1}^{+}$ is calculated as follows.

4 $${s}_{t+1}^{+}=\mathrm{(1}-\alpha )\cdot {({M}^{+})}^{T}\cdot {s}_{t}^{+}+\alpha \cdot d,$$

where (*M* ^+^)^*T*^ is the transpose of the normalization of the positive similarity matrix *M* ^+^, and the *i* ^*th*^ entry ${s}_{t+1}^{+}(i)$ captures the positive probability of the walker teleporting from *u* to *v*_*i*_ at the time *t* + 1.

Iterate the Eq. 4 until *s* ^+^ is convergent. Suppose when the iteration has been completed, the stable state is ${\pi }^{+}=({\pi }_{1}^{+},\ldots ,{\pi }_{m}^{+})$, then *π* ^+^ satisfies *π* ^+^ = (1 − *α*) ⋅ (*M* ^+^)^*T*^ ⋅ *π* ^+^ + *α* ⋅ *d*. In this situation, the *i* ^*th*^ entry of *π* ^+^ denotes the conditional positive probability that the node *u* teleports to *v*_*i*_ when *u* belongs to the *k* ^*th*^ community.

Similarly, we calculate the negative similarity based on negative links by Eq. 5.

5 $$Simila{r}^{-}({v}_{i},{v}_{j})=\frac{|{{\rm{\Gamma }}}_{{v}_{i}}^{-}\cap {{\rm{\Gamma }}}_{{v}_{j}}^{-}|}{|{{\rm{\Gamma }}}_{{v}_{i}}^{-}|\cup |{{\rm{\Gamma }}}_{{v}_{j}}^{-}|},$$

where ${{\rm{\Gamma }}}_{{v}_{i}}^{-}$ (${{\rm{\Gamma }}}_{{v}_{j}}^{-}$) is the negative neighborhood of *v*_*i*_ (*v*_*j*_), the member of which is connected with *v*_*i*_ (*v*_*j*_) by a negative link.

The negative similarities between nodes are normalized to get the transition matrix *M* ^−^. Suppose ${s}_{t}^{-}$ represents the conditional probability that *u* is away from *v*_*i*_ when *u* belongs to the *k* ^*th*^ community at the time *t*. We also calculate the initial negative probability vector ${s}_{0}^{-}$, the *i* ^*th*^ entry of which is the normalization of the negative similarity between *v*_*i*_ and *u*, i.e., ${s}_{0}{(i)}^{-}=\frac{Simila{r}^{-}({v}_{i},u)}{{\sum }_{{v}_{i}}Simila{r}^{-}({v}_{i},u)}$. Then, ${s}_{t+1}^{-}$ could be calculated by Eq. 6.

6 $${s}_{t+1}^{-}=\mathrm{(1}-\alpha )\cdot {({M}^{-})}^{T}\cdot {s}_{t}^{-}+\alpha \cdot d\mathrm{.}$$

Iterate the Eq. 6. When the iteration has been completed, ${\pi }^{-}=({\pi }_{1}^{-},\ldots ,{\pi }_{m}^{-})$ denotes the stable state, where ${\pi }_{i}^{-}$ represents the conditional negative probability that *u* is away from *v*_*i*_ when *u* belongs to the *k* ^*th*^ community.

Next, the node *u* has an average conditional positive probability *p* ^+^(*u* →  *Y*_*j*_|*u* ∈ *G*_*k*_) to teleport to a community *Y*_*j*_ when *u* is connected to the nodes in the *k* ^*th*^ community. Specifically, *p* ^+^(*u* → *Y*_*j*_|*u* ∈ *G*_*k*_) is the mean value of the conditional probabilities and represents *u* teleports to all nodes in *Y*_*j*_ in the graph *G*_*k*_ (Eq. 7).

7 $${p}^{+}(u\to {Y}_{j}|u\in {G}_{k})=mean\{{\pi }_{i}^{+}|{v}_{i}\in {V}_{j}\},$$

where *V*_*j*_ is the node set of the community *Y*_*j*_.

Similarly, *u* also has an an average conditional negative probability *p* ^−^(*u* → *Y*_*j*_|*u* ∈ *G*_*k*_) to be away from *Y*_*j*_ when *u* is connected to the nodes in the *k* ^*th*^ community (Eq. 8).

8 $${p}^{-}(u\to {Y}_{j}|u\in {G}_{k})=mean\{{\pi }_{i}^{-}|{v}_{i}\in {V}_{j}\},$$

The probability that *u* belongs to the *k* ^*th*^ community is based on the positive similarity between *u* and a node in the *k* ^*th*^ community, which is calculated as Eq. 9. We also calculate the probability that *u* does not belong to the *k* ^*th*^ community as Eq 10.

9 $${p}^{+}(u\in {G}_{k}))=avg\{Simila{r}^{+}(u,{v}_{i})|\forall {v}_{i}\in {V}_{k^{\prime} }\mathrm{\}.}$$

10 $${p}^{-}(u\in {G}_{k}))=avg\{Simila{r}^{-}(u,{v}_{i})|\forall {v}_{i}\in {V}_{k^{\prime} }\mathrm{\}.}$$

Finally, the positive probability for the node *u* to teleport to or the negative probability for *u* to be away from a community *Y*_*j*_ is calculated based on the theorem of total probability by Eqs 11 and 12.

11 $${p}^{+}(u\to {Y}_{j})=\sum _{k\mathrm{=1}}^{q}[{p}^{+}(u\to {Y}_{j}|u\in {G}_{k})\times {p}^{+}(u\in {G}_{k})],$$

12 $${p}^{-}(u\to {Y}_{j})=\sum _{k\mathrm{=1}}^{q}[{p}^{-}(u\to {Y}_{j}|u\in {G}_{k})\times {p}^{-}(u\in {G}_{k})],$$

where *p* ^+^(*u* → *Y*_*j*_|*u* ∈ *G*_*k*_) is the average conditional positive probability for *u* teleporting to the community *Y*_*j*_ when *u* is connected to the nodes in the *k* ^*th*^ community, while *p* ^−^(*u* → *Y*_*j*_|*u* ∈ *G*_*k*_) is the average conditional negative probability for *u* being away from to *Y*_*j*_ on the same condition.

The algorithm to calculate the positive and negative probability of a node belonging to each community is described in Table 6. If a node is more likely to be in a community than to be away from the community, then it will be added into the community. Otherwise, it could not be added into any current communities. In this situation, the node could be considered as a new important node, and a new initial community which includes the new important node as well as its close neighbours may be detected. If a new initial community has been detected, then the number of the current communities plus one, and the above procedures are repeated to add nodes into communities; If a new initial community could not be found, *u* will be added to the most likely community by the tightness between *u* and a community *Y*_*j*_ (*j* = 1, …, *q*) as Eq. 13.

13 $$T(u,{Y}_{j})=\frac{nu{m}_{1}}{nu{m}_{2}},$$

where *num*_1_ denotes the number of nodes which have positive connections with the node *u* in the community *Y*_*j*_, and *num*_2_ is the number of nodes in the community *Y*_*j*_. The node is added to the community which has the largest tightness with it.

**Table 6:** Algorithm 1.

*Input*	Node-set *V*; node *u*; community *Y*_*k*_.
	/* The nodes in *V* are those within current communities. */
*Output*	*P* ^+^(*u*_*i*_ → *Y*_*k*_) means the positive probability of *u*_*i*_ belonging to *Y*_*k*_;
	*P* ^−^(*u*_*i*_ → *Y*_*k*_) means the negative probability which represents *u*_*i*_ is away from *Y*_*k*_
Step 1	Construct the graph *G*_*k*_ by Eq. 2.
	/* *G*_*k*_ represents the graph of the *k* ^*th*^ random walk process. */
Step 2	Calculate the positive similarity matrix *Similar* ^+^ and the negative similarity matrix *Similar* ^−^ by Eqs 3 and 5;
	Normalize both *Similar* ^+^ and *Similar* ^−^ to obtain *M* ^+^ and *M* ^−^.
	/* *M* ^+^ and *M* ^−^ are respectively the transition matrix based on positive and negative links.*/
Step 3	Calculate.. and ${s}_{0}^{-}$.
	/*${s}_{0}^{+}$ and ${s}_{0}^{-}$ represent the normalization of the positive and negative similarity between *v*_*i*_ ∈ *V* and *u*. */
Step 4	Calculate $d={(\frac{1}{m},\frac{1}{m},\cdot \cdot \cdot ,\frac{1}{m})}^{T}$. Let *α* = 0.15.
	/* *d* is the transition probability vector and *α* is the teleporting probability.*/
Step 5	Iterate the Eq. 4 until ${s}_{t}^{+}$ is convergent, and let *π* ^+^ to be the convergent ${s}_{t}^{+}$;
	Iterate the Eq. 6 until ${s}_{t}^{-}$ is convergent, and let *π* ^−^ to be the convergent ${s}_{t}^{-}$.
	/* ${s}_{t}^{+}(i)$ means the positive probability of the walker teleporting from *u* to *v*_*i*_ at the time *t*. */
	/* ${s}_{t}^{-}(i)$ means the negative probability that *u* is away from *v*_*i*_ at the time *t*. */
	/* *π* ^+^(*i*) denotes the positive probability of the walker teleporting from *u* to *v*_*i*_. */
	/* *π* ^−^(*i*) denotes the negative probability that *u* is away from *v*_*i*_. */
Step 6	Calculate *p* ^+^(*u* → *Y*_*i*_|*u* ∈ *G*_*k*_) by Eq. 7;
	Calculate *p* ^−^(*u* → *Y*_*i*_|*u* ∈ *G*_*k*_) by Eq. 8.
	/* *p* ^+^(*u* → *Y*_*i*_|*u* ∈ *G*_*k*_) and *p* ^−^(*u* → *Y*_*i*_|*u* ∈ *G*_*k*_) denote an average conditional positive and negative probability
	that *u* teleports to a community *Y*_*j*_ when *u* is connected to the nodes in the *k* ^*th*^ community. */
Step 7	Calculate *p* ^+^(*u* ∈ *G*_*k*_) by Eq. 9;
	Calculate *p* ^−^(*u* ∈ *G*_*k*_) by Eq. 10.
	/*p ^+^(*u* ∈ *G*_*k*_) means the positive probability that *u* is connected to the nodes in the *k* ^*th*^ community. */
	/*p ^−^(*u* ∈ *G*_*k*_) means the negative probability that *u* is connected to the nodes in the *k* ^*th*^ community. */
Step 8	Calculate *p* ^+^(*u* → *Y*_*i*_) = *p* ^+^(*u* → *Y*_*i*_|*u* ∈ *G*_*k*_) × *p* ^+^(*u* ∈ *G*_*k*_) and *p* ^−^(*u* → *Yi*) = *p* ^−^(*u* → *Yi*|*u* ∈ *Gk*) × *p* ^−^(*u* ∈ *Gk*).

### Community optimization

Two or more communities may have a large number of common nodes. That is, these communities may be identical or similar. In this case, the expanded communities should be merged into one community. If communities *C*_*i*_ and *C*_*j*_ satisfy the following formula, then they can be merged into a larger community *C* ^38^.

14 $$\frac{|{C}_{i}\cap {C}_{j}|}{min(|{C}_{i}|,|{C}_{j}|)} > \xi ,$$

where *ξ* is a threshold. Let *ξ* = 0.5, meaning that most members of the small community are in the large community, the two communities can be merged into one.

### Time complexity

The proposed algorithm takes a time complexity of *O*(*dN*) to find local maximum degree nodes in a network, where *d* is the average degree of nodes, and *N* is the number of nodes in the network. At the stage of detecting initial communities, the time used to detect initial communities based on local maximum degree nodes is *O*(*d* ^+^ *p*), where *p* is the number of local maximum degree nodes, and *d* ^+^ is the average positive degree of nodes. In initial situation, there are *p* initial communities at most. At the stage of expanding communities, it needs to calculate the probability that a node teleports to each node in communities based on an iterative formula. It takes a time complexity of *O*(*logm*) in each iteration as stated in ref. 39, where *m* is the number of nodes in the communities. The worst-case complexity for iteration is *O*(*logN*). A small number of nodes (i.e., *h*) which are not in any communities is either in a new community, or to be added to a community based on the tightness. It takes a time complexity of *O*(*p* + *d*) to judge whether a node is in a new community. If a node is in a new community, then the number of initial communities plus one. In the worst case, there are *p* + *h* communities in the stage. Otherwise, it takes the time complexity of *O*((*p* + *h*)*h*) to calculate the tightness between a node and a community. The time complexity of the stage after *p* + *h* iterations is *O*((*p* + *h*) log *N*) + *O*((*p* + *h*)*h*). At the stage of community optimization, it takes a time complexity of *O*((*p* + *h*)^2^) to judge whether two communities should be merged. Therefore, the time complexity of the entire algorithm is *O*((*d* + *p* + *h*)*N*), since *O*(*d* ^+^ *p*) < *O*(*dN*), *O*(*p* log *N*) < *O*(*pN*), *O*(*h* log *N*) < *O*(*hN*) *O*((*p* + *h*)*h*) < *O*(*pN*) + *O*(*hN*) and *O*((*p* + *h*)^2^) < *O*((*p* + *h*)*N*).

### Evaluation measures

Normalized Mutual Information (*NMI*)^14^ and the extended modularity *Q* (*Q*_*signed*_)^21^ are widely used indexes for measuring the performance of community detection algorithms in signed networks. Both of them reflect the detection results from different points of view. Thus, both *NMI* and *Q*_*signed*_ are employed here as indexes to test the detection results.

15 $$NMI({P}_{R},{P}_{F})=\frac{-2\sum _{i}\sum _{j}{X}_{ij}\,\mathrm{log}(\frac{{X}_{ij}N}{{X}_{i\mathrm{.}}{X}_{\mathrm{.}j}})}{\sum _{i}{X}_{i\mathrm{.}}\,\mathrm{log}(\frac{{X}_{i\mathrm{.}}}{N})+\sum _{j}{X}_{\mathrm{.}j}\,\mathrm{log}(\frac{{X}_{\mathrm{.}j}}{N})},$$

where *P*_*R*_ and *P*_*F*_ respectively represent the community partition result obtained by an algorithm and the real community partition; *N* is the number of nodes; *X* is a 2 × 2 matrix, and *X*_*ij*_ is the number of nodes from the real community *i* that also belong to the found community *j*; *X*_.*j*_ = *X*_1*j*_ + *X*_2*j*_; *X*_*i*._ = *X*_*i*1_ + *X*_*i*2_. If the partitioning result *P*_*F*_ is the same as *P*_*R*_, then *NMI*(*P*_*R*_, *P*_*F*_) = 1; if they are completely opposite, then *NMI*(*P*_*R*_, *P*_*F*_) = 0.

16 $$\begin{array}{c}{Q}_{signed}=\frac{1}{2{w}^{+}+2{w}^{-}}\,\sum _{i}\sum _{j}[{w}_{ij}-(\frac{{{w}_{i}}^{+}{{w}_{j}}^{+}}{2{w}^{+}}-\frac{{{w}_{i}}^{-}{{w}_{j}}^{-}}{2{w}^{-}})]\,\delta ({C}_{i},{C}_{j})\end{array}$$

where *w*_*ij*_ is the weight of adjacency matrix, ${{w}_{i}}^{+}({{w}_{j}}^{+})$ denotes the sum of all positive weights of node *v*_*i*_(*v*_*j*_), and ${{w}_{i}}^{-}({{w}_{j}}^{-})$ denotes the sum of all negative weights of node *v*_*i*_(*v*_*j*_). *w* ^+^(*w* ^−^) represents the total positive (negative) strength of the SN, and *C*_*i*_ (*C*_*j*_) represents the community which node *v*_*i*_ (*v*_*j*_) belongs to, and *δ*(*C*_*i*_, *C*_*j*_) is 1 if nodes *v*_*i*_ and *v*_*j*_ are in same community; otherwise *δ*(*C*_*i*_, *C*_*j*_) is 0.

## Electronic supplementary material

Dataset 1
