# Supplementary material for: An algorithm based on positive and negative links for community detection in signed networks
Source: Sci Rep. 2017 Sep 7;7:10874. doi: 10.1038/s41598-017-11463-y (PMC5589891; doi:10.1038/s41598-017-11463-y)
Supplement: Supplementary file 1 — Dataset 1 [file 41598_2017_11463_MOESM1_ESM.doc]

**1 SNM_AFF4**

*Vertices 18

1 1

2 2

3 3

4 4

5 5

6 6

7 7

8 8

9 9

10 10

11 11

12 12

13 13

14 14

15 15

16 16

17 17

18 18

*Arcs

1 2 -1

1 3 1

1 7 -1

1 10 -1

1 12 1

1 14 1

2 1 1

2 4 -1

2 7 1

2 8 -1

2 12 1

2 13 -1

3 1 1

3 2 -1

3 4 -1

3 6 -1

3 13 1

3 16 -1

3 17 1

3 18 1

4 1 -1

4 2 -1

4 5 1

4 6 1

4 11 1

4 14 -1

5 4 1

5 9 1

5 11 1

6 2 -1

6 3 -1

6 4 1

6 5 1

6 7 -1

6 9 1

6 17 -1

7 2 1

7 4 -1

7 6 -1

7 8 -1

7 12 1

7 16 1

8 2 -1

8 3 -1

8 4 1

8 6 1

8 9 1

8 14 -1

9 3 -1

9 5 1

9 8 1

9 12 1

9 17 -1

9 18 -1

10 4 1

10 5 1

10 9 1

10 13 1

11 1 -1

11 2 -1

11 3 -1

11 5 1

11 8 1

11 14 1

12 1 1

12 2 1

12 7 1

13 2 -1

13 5 1

13 6 -1

13 7 1

13 12 -1

13 18 1

14 1 1

14 4 -1

14 8 -1

14 12 1

14 15 1

14 17 -1

15 2 1

15 3 -1

15 4 -1

15 7 1

15 12 1

15 13 -1

16 2 1

16 3 -1

16 4 -1

16 7 1

16 15 1

16 17 -1

17 2 1

17 3 1

17 4 -1

17 6 -1

17 8 -1

17 18 1

18 2 1

18 3 1

18 4 -1

18 8 -1

18 10 -1

18 17 1

**2 SNM_INFL**

*Vertices 18

1 1

2 2

3 3

4 4

5 5

6 6

7 7

8 8

9 9

10 10

11 11

12 12

13 13

14 14

15 15

16 16

17 17

18 18

*Arcs

1 2 1

1 7 1

1 10 -1

1 12 1

1 16 -1

1 18 -1

2 1 1

2 4 -1

2 7 1

2 12 1

2 13 -1

3 1 1

3 2 -1

3 4 -1

3 6 -1

3 13 1

3 17 1

4 1 -1

4 2 -1

4 6 1

4 10 1

4 11 1

4 14 -1

5 2 1

5 3 -1

5 4 1

5 11 1

5 17 -1

5 18 -1

6 2 1

6 4 1

6 7 -1

6 9 1

6 17 -1

6 18 -1

7 2 1

7 3 -1

7 4 -1

7 6 -1

7 8 -1

7 12 1

7 16 1

7 17 -1

8 1 1

8 4 1

8 9 1

8 11 1

8 12 -1

8 13 -1

8 16 -1

9 1 1

9 2 1

9 4 1

9 12 -1

9 17 -1

9 18 -1

11 4 1

11 5 1

11 7 -1

11 9 1

11 17 -1

11 18 -1

12 1 1

12 2 1

12 7 1

13 2 -1

13 5 1

13 6 -1

13 7 1

13 11 1

13 12 -1

14 1 1

14 2 1

14 4 -1

14 12 1

14 13 -1

14 15 1

14 17 -1

14 18 -1

15 2 1

15 3 -1

15 4 -1

15 7 1

15 13 -1

15 14 1

16 2 1

16 3 -1

16 4 -1

16 7 1

16 11 -1

16 15 1

17 2 1

17 3 1

17 4 -1

17 6 -1

17 8 -1

17 18 1

18 2 1

18 3 1

18 4 -1

18 6 -1

18 11 -1

18 17 1

3 Truth partition

1 1 2 0 0 0 1 0 0 0 0 1 2 1 1 1 2 2
